# Immunotherapy and targeted therapy for lung cancer: Current status and future perspectives

**DOI:** 10.3389/fphar.2022.1035171

**Published:** 2022-11-28

**Authors:** Bilal Zulfiqar, Asim Farooq, Shahzina Kanwal, Kashif Asghar

**Affiliations:** ^1^ Griffith Institute for Drug Discovery, Griffith University, Brisbane, QLD, Australia; ^2^ Department of Clinical Research, Shaukat Khanum Memorial Cancer Hospital and Research Centre, Lahore, Pakistan; ^3^ Institute of Molecular Physiology at Shenzhen Bay Laboratory, Shenzhen, China; ^4^ Department of Basic Sciences Research, Shaukat Khanum Memorial Cancer Hospital and Research Centre, Lahore, Pakistan

**Keywords:** immunotherapy, NF-κB, immunosurveillance, lung cancer, targeted therapy

## Abstract

Lung cancer has the highest incidence of morbidity and mortality throughout the globe. A large number of patients are diagnosed with lung cancer at the later stages of the disease. This eliminates surgery as an option and places complete dependence on radiotherapy or chemotherapy, and/or a combination of both, to halt disease progression by targeting the tumor cells. Unfortunately, these therapies have rarely proved to be effective, and this necessitates the search for alternative preventive approaches to reduce the mortality rate of lung cancer. One of the effective therapies against lung cancer comprises targeting the tumor microenvironment. Like any other cancer cells, lung cancer cells tend to use multiple pathways to maintain their survival and suppress different immune responses from the host’s body. This review comprehensively covers the role and the mechanisms that involve the nuclear factor kappa-light-chain-enhancer of activated B cells (NF-κB) in lung adenocarcinoma and methods of treating it by altering the tumor microenvironment. It focuses on the insight and understanding of the lung cancer tumor microenvironment and chemokines, cytokines, and activating molecules that take part in angiogenesis and metastasis. The review paper accounts for the novel and current immunotherapy and targeted therapy available for lung cancer in clinical trials and in the research phases in depth. Special attention is being paid to mark out single or multiple genes that are required for malignancy and survival while developing targeted therapies for lung cancer treatment.

## Highlights

• The tumor microenvironment is intricate and complex and involves a wide variety of chemokines and cytokines.
• Disease progression is promoted in the tumor environment, resulting in inflammatory responses *via* the activation of NF-κB.
• With the development of new targeted therapies, molecular-based therapies have extended their spectrum beyond EGFR, VEGFR, and HER2/neu receptors to the receptor tyrosine kinases (RTKs).
• It is also imperative to evaluate optimal combinatorial approaches, optimal drug sequencing, and redefining and streamlining clinical trials.

## Introduction

Lung cancer has the highest mortality rate compared to all other cancers (Mao et al., 2016; Siegel et al., 2016). In 2012, 1.8 million cases were reported worldwide for lung cancer, which constituted 13% of all cancers reported globally (Torre et al., 2012). In the United States alone, 243,820 new cases of lung cancer were reported, which claimed 162,510 lives (Siegel et al., 2016). The male-to-female ratio is 2:1 and is diagnosed mostly in men aged 60 and above (Mao et al., 2016). Its occurrence is the highest in the regions of Eastern, Central, and Southern Europe for both genders and among women in eastern Asia and North America (Stuber et al., 2008). The major cause of its occurrence is considered to be environmental factors, such as the presence of radon, lead, and other toxic pollutants in the air (Pope et al., 2002). It is also noted that with the prevalence of smoking, particularly in developing countries, the number of cases being reported for lung cancer is proportionally increasing. The mortality rate of lung cancer was recorded to be over 75% with a ratio of 2.2:1 for men to women among people of age 60 years and more (Siegel et al., 2016).

Based on the morphological forms, lung cancer is divided into two main categories, non-small cell lung cancer (NSCLC) and small cell lung cancer (SCLC). The non-small cell lung cancer (NSCLC) is further divided into adenocarcinoma, squamous cell carcinoma, and large cell carcinoma (Cheng et al., 2016). Adenocarcinoma is more prevalent than squamous cell carcinoma in most of the countries around the globe (de Groot and Munden, 2012). It has been observed that a fivefold more number of cases are reported in women as compared to men in Japan, China, and Saudi Arabia (de Groot and Munden, 2012). The reason for the rise in adenocarcinoma cases is linked with cigarette components, use of electronic cigarette (e-cigarette), and environmental factors (Lortet-Tieulent et al., 2014). Before 1979, squamous cell carcinoma was regarded as more prevalent than other forms of cancer and is still a more common type of lung cancer in India, Russia, and the Netherlands (Cheng et al., 2016).

## Tumor microenvironment in lung cancer

**FIGURE 1:**
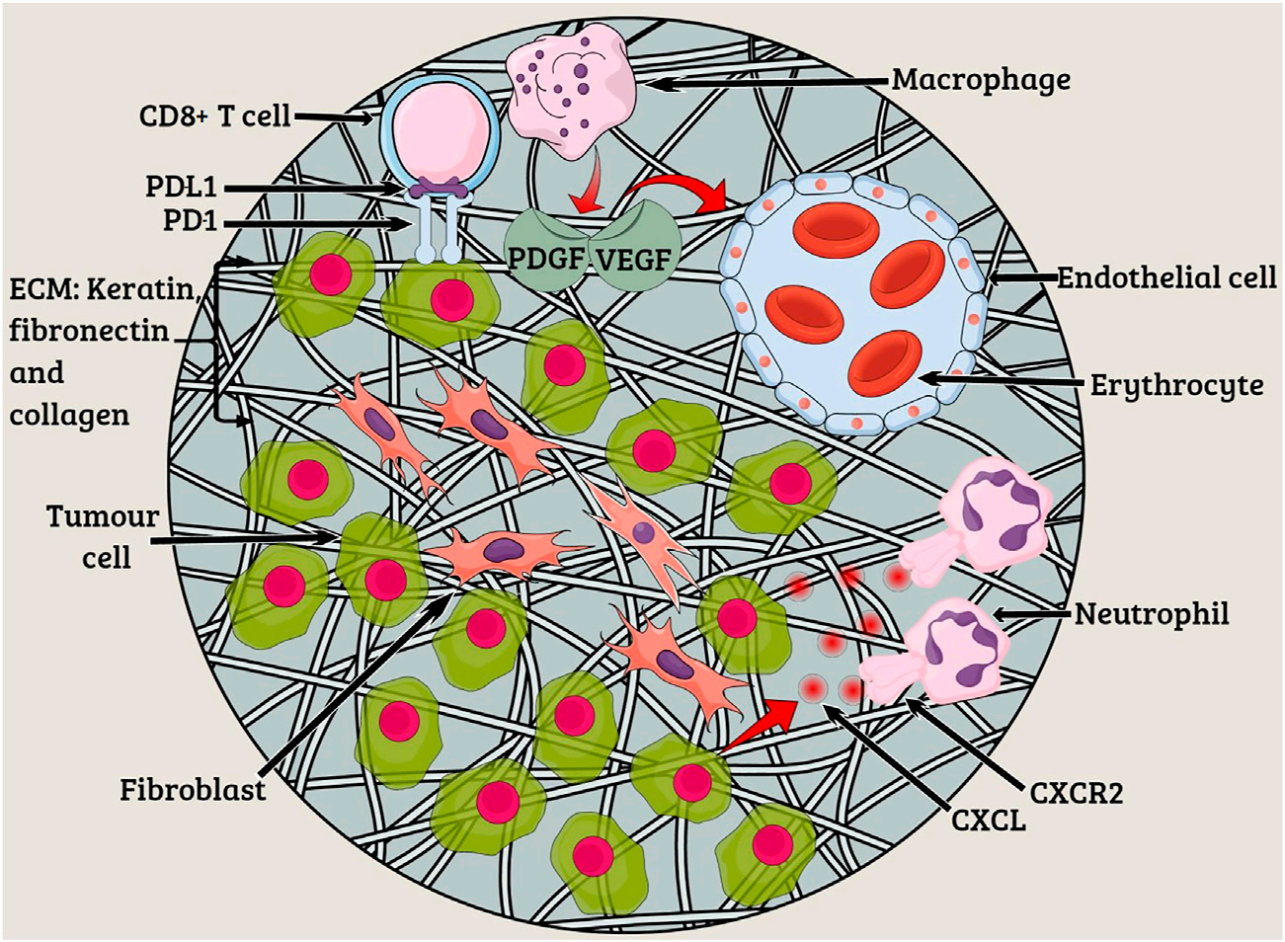
Tumor microenvironment. The characteristics of lung tumors are often determined by fibroblasts, endothelial cells, and myeloid cells existing in the tumor microenvironment. Extracellular matrix (ECM) constituting keratin, fibronectin, and collagen functions to provide structural support to tumor cells. Angiogenesis occurs due to the presence of platelet-derived growth factor (PDGF) and vascular endothelial growth factor (VEGF) at the tumor site. The CXC-chemokine ligand (CXCL) family members bind to the neutrophil receptor CXCR2 to help the tumor cells recruit neutrophils.

Lung adenocarcinoma is a complex disease with a wide array of oncogenes involved along with the cytokines and chemokines, all of which play a significant role in tumor growth and angiogenesis (Grunewald et al., 2006; Li et al., 2011) (Figure 1). The study of cell and molecular biology of lung cancer has emanated from the circuit pathways comprising different key factors that play critical roles in the development of a full-fledged lung cancer. Among these factors, several factors have also been studied for their role at the genetic and epigenetic level and, thus, are considered important for carcinogenesis and metastasis. A variety of compounds/drugs have been developed to specifically target farnesyltransferase, epidermal growth factor receptor (EGFR), and vascular endothelial growth factor receptor (VEGFR). These compounds/drugs have shown encouraging results in clinical trials (Gore et al., 2000; Morabito, 2016).

Signal transduction pathways that are responsible for cell proliferation and survival include mitogen-activated protein kinases (MAPKs) (Vicent et al., 2004), a serine/threonine kinase AKT (Brognard et al., 2001), and NF-κB (Jones et al., 2000), which are hijacked or altered to facilitate these functions and maintain tumorous growth.

NF-κB is the key mediator of the tumor microenvironment and is constitutively active in different tumor cells. The key signaling pathway, involved in a wide array of functions, is activated in the case of lung adenocarcinoma both in murine models and humans (Karin and Greten, 2005; Karin, 2006; Meylan et al., 2009; Basseres et al., 2010). The T-cell infiltration in the tumors is associated with immunosurveillance and tumor immunoediting, thus increasing the patient quality of life and survival rate. NF-κB has been a potent factor involved in protumor responses by boosting and recruiting the immunosuppressive cells, which include the regulatory T cells (Tregs) and myeloid dendritic cells (mDCs). These cells activate and release chemokines and cytokines along with the growth factors such as VEGF that initiate tumor growth and angiogenesis. Mutations in NF-κB enhance angiogenesis and metastasis by ultimately inducing mutations. Type 1 interferons including IFN alpha and beta and interferon gamma have pivotal roles in cancer immunosurveillance and priming of T cells in tumors. The effector functions of interferon gamma play a significant role in cancer immunoediting and natural killer cell activation. T-cell priming also activates the complement system and mediates the antitumor responses. A crosstalk at the molecular level between the interferon and the NF-κB pathway plays a significant role in the tumor microenvironment (Muthuswamy et al., 2012; Hopewell et al., 2013).

**FIGURE 2:**
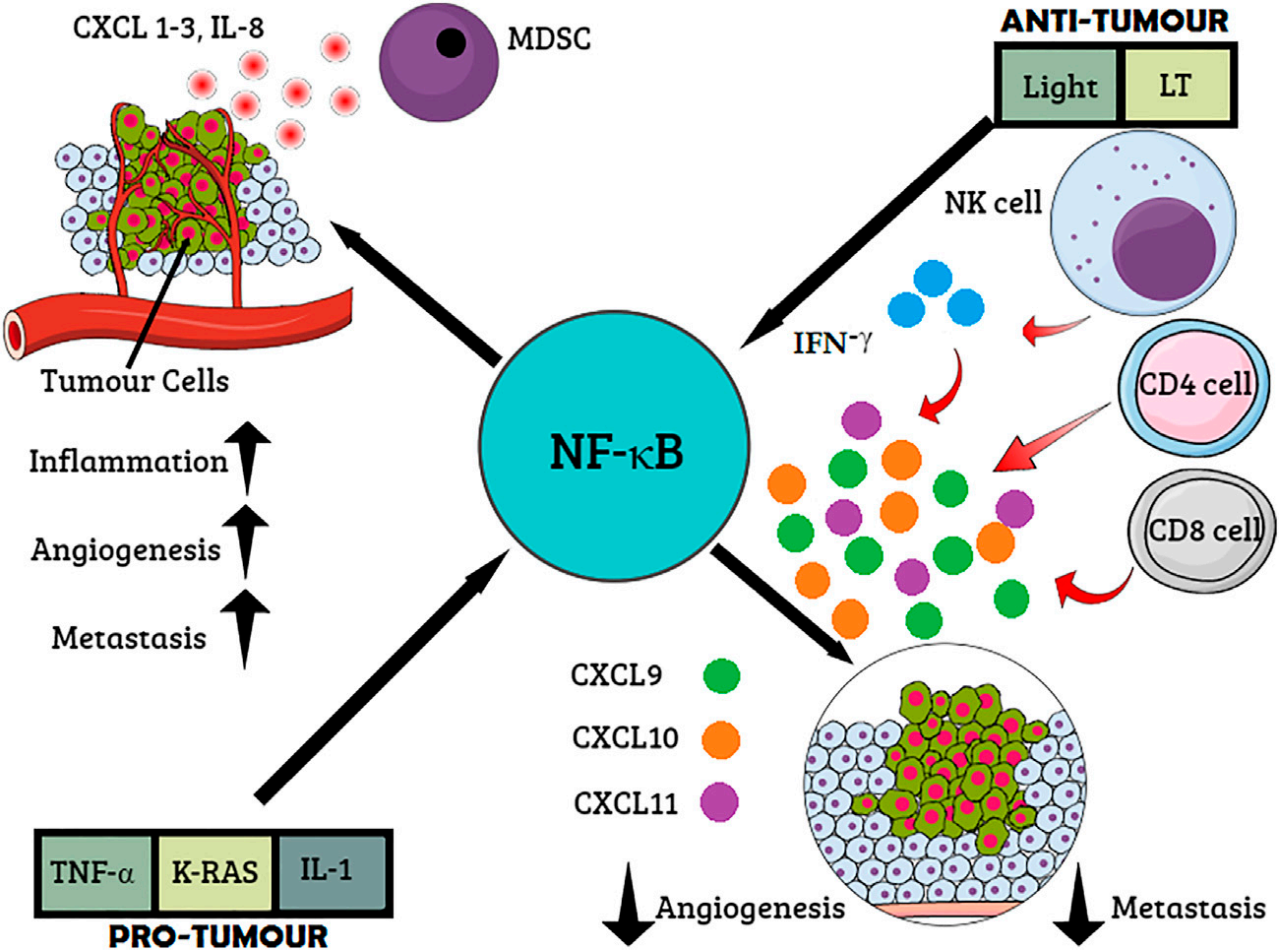
Role of NF-κB cancer immunosurveillance. Proinflammatory cytokines and oncogenes activate NF-κB resulting in the expression of proinflammatory mediators such as chemokine CXCL 1-3, interleukin-8 (IL-8), and (C-X-C motif) ligand. The recruitment of myeloid-derived suppressor cells (MDSCs) inhibits the antitumor response. Inflammation, angiogenesis, and metastasis are stimulated *via* multiple chemokines. On the other hand, interferon (IFN)-γ produced by T cells or natural killer (NK) cells stimulates the secretion of CXCL9–11, which, in turn, inflicts antiangiogenic and antimetastatic effects.

**FIGURE 3:**
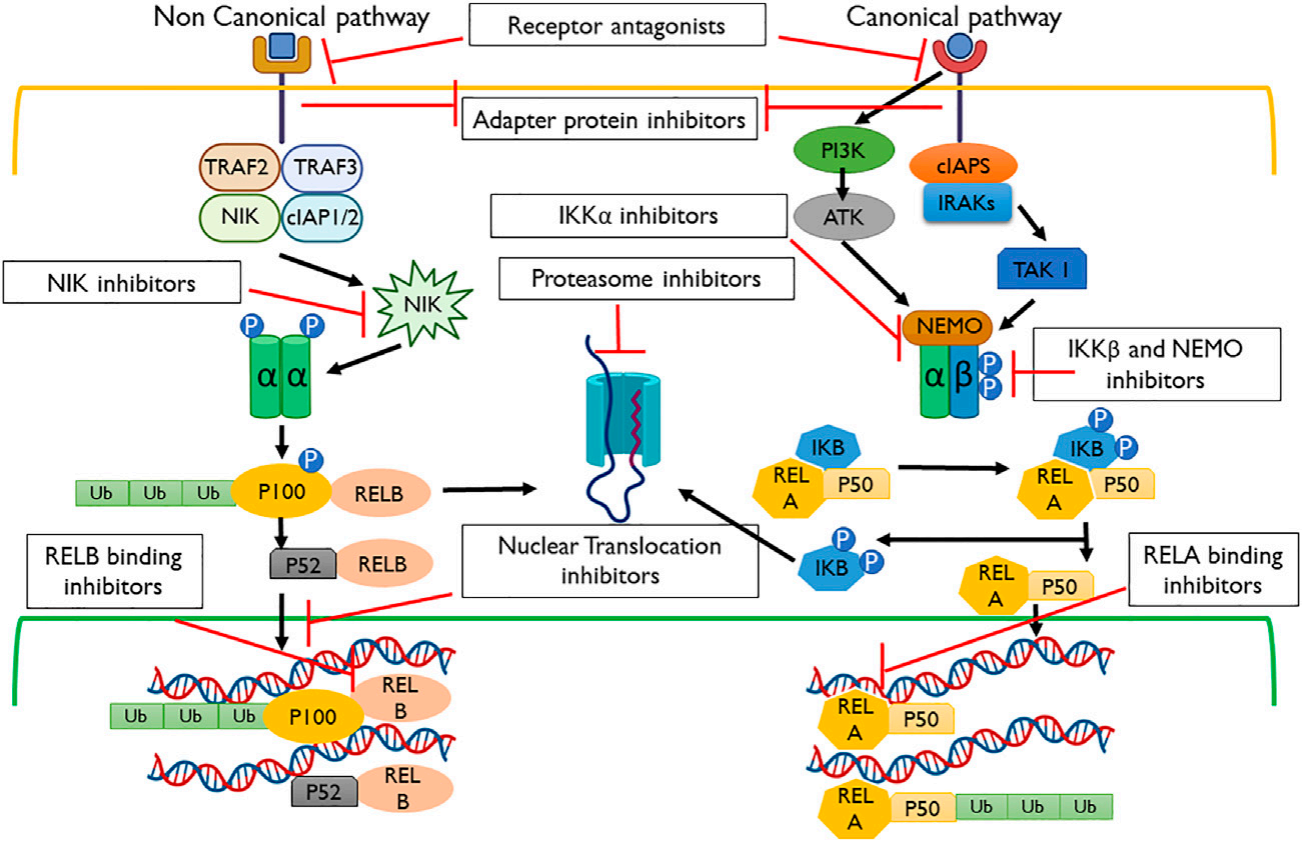
Pathways and inhibitors for NF-κB activation. These signaling cascades are modulated within the non-canonical and canonical pathways using the receptor, adapter protein, IKKα, proteasome, NF-κB-inducing kinase (NIK), nuclear translocation, REL-associated protein A (RELA), REL-associated protein B (RELB), and NF-κB essential modulator (NEMO) inhibitors.

The antitumor responses of the NF-κB trigger signaling cascade result in T-cell recruitment at the tumor site, leading to tumor regression and activation of chemokines and cytokines possessing the C–C motif and CCL2, respectively (Xia et al., 2014). NF-κB mediates both protumor and antitumor responses along with interferon activation and T-cell activation (Figure 2) (Zhang et al., 2021). The roles of different mediators, which include Toll-like receptors, lymphotoxin beta (LTB), intercellular adhesion molecule 1 (ICAM1), interferon beta, chemokines, and cytokines, are linked to the NF-κB activation and promotion of tumor regression, leading to better disease prognosis (Liu et al., 2017). Under the action of the aforementioned mediators and immunomodulatory genes, NF-κB regulation in inflammatory and immune responses opens up new avenues of research and a better prognosis of lung adenocarcinoma, which can be solved by immunotherapy. The Pathways and inhibitors for NF-κB activation have been shown in Figure 3.

T-cell proliferation is regulated by the cytotoxic T-lymphocyte-associated antigen 4 (CTLA-4) and programmed death cell receptor 1 (PD-1). They are associated with NF-κB. The tumor microenvironment is complex and consists of intricate crosstalk of different signaling factors, chemokines, cytokines, and genes that need investigation and focus to target the soil rather than the seed of the tumor cells (Muthuswamy et al., 2012).

Disease progression is promoted in the tumor environment, resulting in inflammatory responses *via* the activation of NF-κB (Akca et al., 2011). It has recently been found that in tumor cells, T cell-mediated immune response is also regulated by the activation of NF-κB, hence actively participating in cancer immunosurveillance (Zhu et al., 2016).

### Domains of the nuclear factor kappa-light-chain-enhancer of activated B cells

Five family members of NF-κB have been identified in mammalian cells. These are RelA (REL-associated protein A) (p65), RelB (REL-associated protein B), c-Rel, NF-κB1 (p50/p105), and NF-κB2 (p52/p100). All of these contain an N-terminal domain called RHD (Rel homology domain) that makes them a member of this family and is used in forming a homo/heterodimer that can bind to the DNA (Hayden and Ghosh, 2004)*.* p65, RelB, and c-Rel also contain a domain called the trans-activator domain (TAD), through which they bind with p50 or p52 members, resulting in their activation in a trans manner. p50 and p52 lack TAD on their C-terminals. Also, the p50 and p52 homodimers are transcription repressors and, in this configuration, develop a threshold for NF-κB activation (Ghosh et al., 1998).

However, in a normal physiological condition, NF-κB dimers are present in cells but are withheld within the cytoplasm by their inhibitors that mask their NLS (nuclear localization sequence) domain. These inhibitors are considered to be specific for each member of the family that includes IκBα, IκBβ, IκBγ, IκBϵ, and BCL-3, and they keep a tight check on the activation of NF-κB pathways (Lin et al., 2010).

### Pathways for the nuclear factor kappa-light-chain-enhancer of activated B cells

NF-κB is a multifunctional transcription factor that can be activated *via* various extracellular signals generated due to genotoxic or endoreticulum stress, including growth factors, cytokines, carcinogens, intracellular stimuli, and tumor promoters (Tak and Firestein, 2001).

A canonical pathway can be activated by proinflammatory growth factors, microbial infections, and cytokines including TNFα. TNFα on binding with TNFR1 (TNFα receptor 1) causes its transmerization leading to the recruitment of several proteins that phosphorylate and activate IKK (IκB kinase complex) (Lin et al., 2010)*.* The IKK complex consists of three subunits involved in its catalytic reactions: IKKα/IKK1, IKKβ/IKK2, and an essential regulatory subunit, IKKγ/nuclear factor-κB essential modulator (NEMO) (Karin, 1999). In the canonical pathway, IKKβ plays an important role as it gets phosphorylated on its serine residues 32 and 36 and results in its ubiquitination and degradation, thereby freeing NF-κB p50, p65, and c-Rel (Karin, 1999). The NLS domains present on these NF-κB molecules are now exposed and modified to allow binding to the DNA or to transcriptional factors such as CBP (cAMP response element-binding protein) (Chen and Greene, 2004).

Also, in the case of DNA damage by radiation and genotoxic agents, the IKKB-NF-κB cascade can be elicited. In this scenario, the pathway is activated by the activation of ATM (ataxia telangiectasia-mutated kinase) that phosphorylates the IKKγ domain bound to a complex called PIDDsome (Tinel and Tschopp, 2004). This complex consists of a receptor-interacting protein (RIP1), p53-induced death domain, and NF-κB essential modulator (NEMO). When NEMO is phosphorylated, it detaches itself from the complex and moves into the cytoplasm, resulting in the transactivation of IKKβ, and this serves as the initiation of the canonical pathway (Lee et al., 2012).

Apart from the aforementioned pathway, cells have a non-canonical pathway involving non-death receptor members of the TNF receptor family (Muppidi et al., 2004). These include the cluster of differentiation 40 (CD40), lymphotoxin beta, and B-cell activating factors (Muppidi et al., 2004). These receptors are activated by their specific ligands, resulting in the stabilization and auto-activation of NIK (NF-κB-inducing kinase), which further phosphorylates the IKKα member of the NF-κB family (Kratz et al., 2016). IKKα, in response to its activation, undergoes a conformational change and cleaves its p100 to produce a functional NF-κB heterodimer containing the newly cleaved p52 and RelB, which is then translocated to the nucleus to act as a functional transcription factor (Kratz et al., 2016).

Canonical and non-canonical pathways are regulated by c-IAP (inhibitor of apoptosis) proteins. These proteins suppress the non-canonical pathway by causing ubiquitination of NIK under normal conditions (Kocab and Duckett, 2016). However, K-Ras can bind to and activate NF-κB through TBK1 (TANK-binding kinase 1) in the non-canonical pathway and contribute to oncogenic K-Ras-mediated lung carcinogenesis. NF-κB can also be activated by the components contained in tobacco; among these, nicotine and methylnitrosamino-1-(3-pyridyl)-1-butanone (NNK) are seen in a panel of NSCLC cell lines (Kim et al., 2016). As in smokers’ lungs, NF-κB is constantly activated, and it is possible that it allows cancer cell proliferation and escape from apoptosis in the very early stage of lung cancer development (Chen, 2005).

## Chemokines and cytokines in the tumor microenvironment

The tumor microenvironment is intricate and involves a wide variety of chemokines and cytokines. In this section, we will discuss these chemokines and cytokines.

### Vascular endothelial growth factor

Vascular endothelial growth factor (VEGF) is the most significant regulator of angiogenesis and is the requisite for the growth and viability of tumors in the microenvironment (Koch et al., 2011). VEGF chemokine induces the expression of the C-X-C motif Chemokine ligand 12 (CXCL12) are chemokines formed by the activation of myofibroblasts and tumor macrophages. CXCL12 chemokines have a high expression of the epidermal growth factor and promotes the formation of new vessels in the tumor cells, consequently increasing the chances of metastasis (Koch et al., 2011). A strong interaction exists between the cell surface receptors and C-X-C chemokine receptor type 4 (CXCR4) in lung cancer (Takahashi, 2011).

### Role of chemokine receptors in non-small cell lung cancer

The CXCR4 chemokine plays a very significant role in non-small cell lung cancer (NSCLC) metastasis and is an important component of the tumor microenvironment (Wu et al., 2010). The high levels of CXCR4 chemokine are investigated using immunohistochemistry. CXCR4 is involved in the role of pleural spaces with its levels correlated with the expression of the CXCL12 chemokine, which is present in the advanced stages of the disease. The CXCL12 chemokine has a high expression on stromal cells, neoplastic cells, and vascular and endothelial cells in the patients suffering from lung adenocarcinoma study of cancer patients at stages I and II (Chen, 2005; Grunewald et al., 2006; Li et al., 2011). CXCL12 expression in NSCLC cells (*in vitro*) indicates the correlation between CXCL12 and CXCR4 chemokines, which induces the extracellular signal-regulated kinase (ERK) pathways and growth-forming factor activation. They are the keynote chemokines associated with tumor growth along with the accessory cells such as the T regulatory cells. These chemokines act in the paracrine and autocrine fashion and attract other growth-promoting and inflammatory cytokines, which mediate the process of angiogenesis and tumor growth (Chen, 2005; Grunewald et al., 2006; Li et al., 2011).

In the later part of this review, we turn our focus to the current and available immunotherapies, anticancer drugs, and vaccines that are available for lung adenocarcinoma.

### Molecular-based targeted therapies for lung cancer

Extensive research is being carried out to pinpoint the key players playing pivotal roles in malignancy and/or cell survival while exploiting this knowledge to develop targeted therapies for lung cancer treatment. As described in Table 1, several new drugs have been developed, which target these specific factors, and their clinical trials have revealed positive and encouraging results (Dunn et al., 2006).

**TABLE 1:** Novel and current immunotherapies available for lung cancer in clinical trials and in the research phases.

Molecule	System	Receptor/target	Mechanism of action	Reference
Erlotinib	Inhibitor	EGFR	Inhibits intracellular phosphorylation of tyrosine kinase-associated EGFR	Kim and Murren (2000)
Gefitinib	Inhibitor	EGFR	Reduces cell proliferation *via* inhibiting EGFR	Ciardiello et al. (2000)
Tipifarnib	Inhibitor	Farnesyltransferase	Inhibits Ras kinase before the kinase pathway becomes hyperactive	Venkatasubbarao et al. (2005)
Lonafarnib	Inhibitor	Farnesyltransferase	Inhibits Ras kinase before the kinase pathway becomes hyperactive	Lee et al. (2004)
Sorafenib	Inhibitor	VEGFR, PDGFR, and Raf family kinases	Induces autophagy, resulting in suppressed tumor growth	Eisen et al. (2006), Gridelli et al. (2007)
Afatinib	Inhibitor	EGFR/HER2 blocker and TK protein inhibitor	Inhibition of HER2, HER4, and EGFR. Afatinib also inhibits transphosphorylation of HER3	Cordier et al. (2011), Wind et al. (2017)
Crizotinib	Inhibitor	ALK protein	Inhibits ALK protein	Forde and Rudin (2012)
Nivolumab	Monoclonal antibody	PD-1 molecule	Binds to the PD-1 molecule and induces programmed tumor cell death	Taneja (2012)
Bevacizumab	Monoclonal antibody	VEGF	Prevents interaction with its receptor by selectively binding to VEGF.	Han et al. (2016)
Cetuximab	Monoclonal antibody	EGF receptor	Inhibition of enhanced apoptosis, cell proliferation, and reduced invasiveness, angiogenesis, and metastasis	Suwa et al. (1999)
Pembrolizumab	Monoclonal antibody	PD-1 molecule	Binds to PD-1 molecule and induces programmed tumor cell death	McDermott and Pembrolizumab (1998)
Oxamflatin	Inhibitor	Histone deacetylase	Inhibits cell differentiation, proliferation, motility, and survival	Kim et al. (1999)
Vorinostat (suberanilohydroxamic acid)	Inhibitor	Histone deacetylase	Inhibits cell differentiation	Xu et al. (2007)
Vandetanib	Inhibitor	VEGF	Inhibits VEGFR, EGFR, and RET tyrosine kinases	Commander et al. (2011)
		EGFR receptor-2 tyrosine kinase		
Cediranib (AZD2171)	Inhibitor	VEGF	Limits the growth of new blood vessels and starves tumor cells	Wedge et al. (2005)

### Epidermal growth factor receptor inhibitors

Epidermal growth factor receptor (EGFR) pathways are mostly observed to be dysregulated in human cancers, attracting researchers for targeted anticancer therapy (Sharma et al., 2007). The noted EGFR family includes the following members, EGFR (epidermal growth factor receptor 1, also known as HER1 or ERBB1), HER2 (ERBB2/NEU or EGFR2), HER3 (ERBB3 or EGFR3), and HER4 (ERBB4 or EGFR4). The EGFR family consists of receptor tyrosine kinase (TK), a transmembrane receptor involved in cellular growth and proliferation (Sharma et al., 2007). Upon binding of the ligand, the EGFR intracellular domain dimerizes and activates the TK domain and its autophosphorylation, which runs an intracellular cascade that leads to the inhibition of apoptosis, while the increase in cellular proliferation, angiogenesis, and invasion ultimately leads to tumor generation and metastasis (Shigematsu and Gazdar, 2006). Of note, mostly EGFR (ERBB1) along with ligands is found overexpressed in NSCLC tumors. It is possible that the members of the EGFR family of receptors can heterodimerize with each other, so in order to identify the pharmacological therapeutic target, it is important to have a robust grip of knowledge about the ERB receptors expressed in tumor cells (Dunn et al., 2006).

Erlotinib and gefitinib are molecular TKIs of EGFR, of which only the former is presently approved for NSCLC treatment in the United States (Miller et al., 2012). Significant improvement was observed in phase III clinical trials of erlotinib along with a placebo given to the patient previously treated antecedently with an advanced NSCLC (Miller et al., 2012). For this study practice, 731 subjects who had previously received one to two chemotherapies were recruited in a ratio pattern of 2:1 in order to administer erlotinib/placebo. The response rate observed was 8.9% and <1% in the erlotinib and placebo categories, respectively.

Gefitinib also responded positively in phase II trials, but its adequate survival rate was not observed in phase III trials. Some researchers theorized that it was because erlotinib was administered at MTD (maximum tolerated dose), while gefitinib was below its MTD (Cataldo et al., 2011). Moreover, the acceptability criteria for both were also different in gefitinib trials, and the patients recruited made progress within 90 days of the previous chemotherapy. Gefitinib is currently provided to a patient who benefits from it or who is involved in clinical trials (Cataldo et al., 2011; Fuertes et al., 2013).

Gefitinib and erlotinib have both been studied in different groups of patients along with cytotoxic chemotherapy, but no overall positive response has been observed (Cataldo et al., 2011). However, it is proposed in some retrospective analytical studies that patients who never smoked may derive benefits from this combination. However, tumor mutation in EGFR and its amplification status are strongly associated with EGFR TKI therapy’s positive response. All of these trials have also revealed that a patient deprived of these features can also respond positively (Sun et al., 2007).

### Kirsten rat sarcoma virus gene mutations and inhibitors

KRAS is a proto-oncogene product that plays a role in the cellular proliferation mechanism. Among the mutations observed in the RAS family, 90% are found in KRAS proteins in smoker NSCLC patients with rare survival (Pao et al., 2005). Normally, EGFR and KRAS mutations are not associated, but KRAS mutations have been observed to develop as a result of resistance to the EGFR therapy at the primary level (Riely et al., 2009). Currently, many agents targeting Kirsten rat sarcoma virus gene (KRAS) pathways at their different steps have been developed and are in clinical trials. Among these, farnesyltransferase inhibitors (FTIs) have been studied; in particular, tipifarnib and lonafarnib are orally available TKIs that are being analyzed in combination with cytotoxic chemotherapy (Kim et al., 2005).

B-Raf proto-oncogene (BRAF) is also found to be an important downregulating agent for the RAS pathway and is considered a balanced therapeutic target (Brose et al., 2002). Sorafenib is an orally available dual-action multikinase inhibitor drug that acts as an antiangiogenic agent and functions as a BRAF inhibitor. Additionally, it inhibits VEGFR and PDGFR (Wilhelm et al., 2008; Scagliotti et al., 2010). Early trials of this drug revealed adequate tolerance as a cytostatic agent and with prolonged disease stabilization. Phase II trials for sorafenib are in progress in previously treated NSCLC patients (Scagliotti et al., 2010). MEK inhibitors have recently been developed, which downregulate the RAS/RAF pathway reaction. The preclinical and initial clinical trials have revealed their covenant antitumor activity in NSCLC patients, while phase II studies are in progress (Brose et al., 2002; Wilhelm et al., 2008; Fuertes et al., 2013).

### Histone deacetylase inhibitors

Histone deacetylase (HDAC) inhibitors have been observed to arrest cellular differentiation, growth, and apoptosis in tumors acquired in cell culture in melanoma, leukemia, prostate, breast, ovarian, and lung cancers (Marks et al., 2004). Many HDAC inhibitors have been observed in arresting tumor proliferation in cancerous animal models. The inhibitors include depsipeptide MS-27-275, oxamflatin, and suberanilohydroxamic acid (SAHA) (Kumar et al., 2015). It has been observed that SAHA inhibits tumor growth in methylnitrosourea-induced mammary carcinoma (Zhu et al., 2013). SAHA and its second hybrid polar hydroxamic acid-based HDAC inhibitor have been approved for clinical trials (Sun et al., 2007).

### Angiogenesis inhibitors

High expression of vascular endothelial growth factor receptors (including all family receptors VEGF-A, -B, -C, -D, and -E) is observed in NSCLC patients and is strongly related to tumor progression and poor prognosis (Smith et al., 2010). Several molecular therapeutic agents designed to target these receptors are in clinical and preclinical trials (Batchelor et al., 2010). The monoclonal antibodies against these receptors are extensively studied (Perren et al., 2011).

A monoclonal antibody named bevacizumab, possessing the equal potential to bind with all VEGF isoforms, gained success in clinical trials (Perren et al., 2011). Recently, different studies have revealed that the addition of carboplatin and paclitaxel to bevacizumab showed encouraging survival benefits in first-line treatment of advanced nonsquamous NSCLC patients (Dahlberg et al., 2010). In combination with other therapies, bevacizumab is still in trials for lung cancer treatment.

VEGFR TKIs are molecular inhibitors designed to target the ATP pocket of TK in the intracellular domain of VEGFR that leads to the blockage of its cellular cascade (Choueiri et al., 2010). Zactima is an orally available molecular inhibitor that is capable of binding to VEGFR2 to a greater extent as compared to EGFR (Robert, 2010). The recent use of zactima in combination with docetaxel in phase II trials on patients with advanced NSCLC has revealed an improved and progression-free survival rate as compared to only docetaxel therapy and has been approved for phase III trials recently (Robert, 2010). AZD2171, along with carboplatin and paclitaxel, showed an efficient antitumor activity as second-line therapy and is well-tolerated in advanced NSCLC patients (Ramalingam et al., 2010). The phase II/III trials of this combination therapy are also in progress (Dunn et al., 2006).

### New targets and perspectives

With the development of new targeted therapies, molecular- based therapies have extended their spectrum beyond EGFR, VEGFR, and HER2/*neu* receptors to the receptor tyrosine kinases (RTKs). The most important RTK is the platelet-derived growth factor (PDGF), which is an attractive target for oncology field researchers. Its expression has been observed in fibroblasts, smooth muscles, the brain, testes, and kidneys (Clark, 2013). The overexpression of PDGF and PDGFR has also been observed in a large proportion of glioblastoma tumors. It establishes an autocrine stimulatory loop that is thought to be important in tumor establishment and proliferation (Zarghooni et al., 2010). The same loop is diagnosed in various cancers like meningioma, neuroendocrine cancer, ovarian, pancreatic, gastrointestinal, prostate, and lung cancers. As far as the inhibitors of PDGF/PDGFR are concerned, CDP680 (cell tech) is under phase I trials (Raica and Cimpean, 2010), whereas clinical trials for SU101 are stopped at phase III due to their acute pharmacokinetic variability (Raica and Cimpean, 2010). In addition to the RTK-targeted therapy, many other kinases in the cytoplasm are thought to play a major role in cell cycle regulation, gene expression, cell death, and metabolism. These kinases are considered an important joint for these pathways and could be important molecular targets for anticancer therapy (Marks et al., 2001; Heist and Christiani, 2009). One of the very first anti-CTLA-4 blocking antibodies ipilimumab (IgG1) was tested and approved for melanoma cancer patients (Phan et al., 2003). Tremelimumab (IgG2) also belongs to the same pharmacological class, and both these monoclonal antibodies are undergoing clinical trials for NSCLC patients. T-lymphocyte-associated protein 4 (CTLA-4) (CD152) belongs to the B7/CD28 family that inhibits T-cell functions (Chan et al., 2014). It is regarded as an immune checkpoint receptor as it diminishes signaling through CD28, which induces immunosuppression (Rudd et al., 2009). CTLA-4 is expressed on tumor cells, exhausted conventional T cells, and infiltrating Tregs (Huang et al., 2016). Apart from its involvement in immunosuppression, its role in disease progression is still unknown.

### Indoleamine 2,3-dioxygenase

Indoleamine 2,3-dioxygenase (IDO) is an immunosuppressive enzyme that mediates the catabolism of tryptophan. IDO is produced both in tumor cells and antigen-presenting cells (Platten et al., 2012).

IDO induces immune tolerance in the tumor microenvironment through the depletion of tryptophan, and its toxic catabolites subsequently inhibit T-cell proliferation and T-cell immune response (Hwu et al., 2000). Furthermore, IDO has the ability to inhibit T-cell immunity by inducing the differentiation and maturation of Tregs (Nakamura et al., 2007). IFN-γ is the most potent inducer of IDO (Basu et al., 2006). NF-κB transcription factors are crucial for the expression of proinflammatory cytokines in DCs (Ouaaz et al., 2002) and have been implicated in IDO induction (Du et al., 2000). It has been recognized that IDO emerging from tumors has the capacity to inhibit antitumor immunity and promote metastasis (Uyttenhove et al., 2003; Sakurai et al., 2005). Smith et al. (2012) observed that IDO is involved in the development of lung cancer metastasis in a mouse model. Chung et al. (2014) identified that the IDO activity contributes to interferon-γ-induced apoptosis in NSCLC. Karanikas et al demonstrated that IDO is not only contributing to tumor immune escape but may also mediate the immune conditioning of the peri-tumoral lung area (Karanikas et al., 2007). A comprehensive study published by Creelan et al explicated that IDO may partake in the resistance of NSCLC to therapy, and further studies will be necessary to investigate the antineoplastic effects of IDO inhibitors, such as 1-methyl-D-tryptophan (D-1MT) (Creelan et al., 2013). Yang et al. (2013) established that IDO inhibitors reduced the number of regulatory T cells and presented therapeutic activities against Lewis lung cancer in a mouse model. Astigiano et al. (2005) suggested that IDO has the potential to be used as a prognostic marker in NSCLC. Another conclusive study published by Schafer et al. (2016) pointed out that IDO inhibitors, as an adjuvant therapy, can promote antitumor immunity against lung cancer. Further studies will be required to investigate the immunosuppressive role of IDO in lung cancer, in order to facilitate the development of efficient anticancer immunotherapy.

### Non-small cell lung cancer stem cells

The aggressiveness of non-small cell lung cancer and resistance to different drugs depicted its heterogeneity and increased the plausibility of stem cell presence. The gross root hindrance for taking control of cancerous cells is to stop uncontrolled proliferation, which is the hallmark of undifferentiated/stem cells. Moreover, cancer stem cells have the ability to hideout in the dormant/quiescent phase of growth, which can also be contributed by stem cells, and this capability acts as one of the devils causing intrinsic and acquired drug resistance. Several studies demonstrated the plasticity of different cancer cells including NSCLC (Gupta et al., 2009; Leung et al., 2010; Akunuru et al., 2012; Sterlacci et al., 2014). A number of studies observed a correlation between metastatic invasion and stemness of NSCLC, reviewed by Gottschling et al. (2012). An epithelial-to-mesenchymal transition (EMT), which is considered another hallmark of cancer cells, has also been found to be associated with stem cell presence. NSCLC possessing stem cells showed low sensitivity to different cancer drugs (Perona et al., 2011). Moreover, ionization surviving cancers exhibit the mesenchymal phenotype with a higher expression of stem cell markers, for example, CD44 and CD24 (Gomez-Casal et al., 2013; Sterlacci et al., 2014). The aforementioned studies are indicating the significance of stem cell studies in prognosis and in stem cell therapeutics. Therefore, we need to focus on the exploitation of stem cells in NSCLC as these hidden culprits need to be targeted for effective therapy.

### Development of immunotherapy for non-small cell lung cancer

The future of immunotherapy lies with the perpetual research in tumor immunology (Mikulski et al., 1979). In 1991, 16 patients with metastatic NSCLC were treated with IL-2 in combination with TNF- α. The results of phase-I clinical trials showed that low doses of TNF-α and IL-2 mediate tumor regression in advanced-stage NSCLC patients (Yang et al., 1991). In 1992, Jansen et al. (1992) concluded that a combination of IL-2 and IFN-α was ineffective for the treatment of NSCLC patients. A study published in 1993 stated that the administration of recombinant IL-2 therapy resulted in increased circulating immune cells with a potential antitumor activity (Scudeletti et al., 1993). In 1995, Ratto et al. (1995) showed that adoptive immunotherapy might be given to patients with stage-III NSCLC.

In 2001, Palmer et al. (2001) conducted a phase-I clinical trial on the BLP25 liposomal vaccine and concluded that the vaccine generated an immune response in lung cancer patients. In 2005, Ishikawa et al. (2005) conducted a phase-I clinical trial of α-galactosylceramide (KRN7000)-pulsed dendritic cells and concluded that it was well tolerated and could be administrated safely in patients with advanced diseases. In 2006, telomerase peptide vaccination was shown to induce immunogenic responses in patients with NSCLC, and further clinical studies of these peptides were warranted (Brunsvig et al., 2006). In 2008, Wu et al. (2008) concluded that the combination of chemotherapy with cytokine-induced killer cells could ameliorate patients’ cellular response and help patients in recovery. In a different study, Li et al. (2009) stated that dendritic cell-activated cytokine-induced killer cells enhanced the outcomes of chemotherapy in NSCLC patients. A group of researchers conducted a study of adoptive immunotherapy in patients with NSCLC and suggested that T-cell immunotherapy might be safe and feasible for patients with recurrent NSCLC (Nakajima et al., 2010).

In 2011, Jensen et al. (2011) proposed that radioimmunotherapy with cetuximab was particularly efficacious in elderly patients with various comorbidities. In 2012, Pan et al. (2012) carried out a study on the monoclonal antibody NJ001 and concluded that it selectively reacted to NSCLC and exhibited an antitumor activity. Wang et al. (2014) indicated that haploidentical cytokine-induced killer cells were effective in prolonging the survival of NSCLC patients. In 2015, Pujol et al. (2015) showed that MAGE-A3 induced a specific immune response in resected and unresected NSCLC patients. In 2016, Rodriguez et al. (2016) conducted a study on CIMAvax-EGF (an epidermal growth factor vaccine) and showed its efficacy in the control of EGF-dependent NSCLC tumors. A pilot study was carried out to analyze the efficacy of an autologous tumor-derived autophagosome vaccine (DRibbles), and it was reported that the vaccine given in combination with GM-CSF was capable of inducing an immune response against tumor cells (Sanborn et al., 2017). Zhao et al. (2018) showed that blocking PD-1 in combination with retronectin-activated cytokine-induced killer cells was valuable in NSCLC patients with advanced diseases. In 2019, Koopman et al. (2019) demonstrated that enapotamab vedotin (an AXL-specific antibody–drug conjugate) shows promising therapeutic potential in NSCLC. Recently, Martin et al. (2020) have showed that nivolumab is a promising antibody for NSCLC patients. Another study conducted by Arrieta et al. (2020) concluded that pembrolizumab in combination with docetaxel improved the overall response rate and progression-free survival in patients with advanced diseases.

## Conclusion

It has been established up until now that the tumor microenvironment plays a major role in tumor formation, survival, and in immune evasion in lung cancer. NF-κB plays a dual function of either tumor clearance or tumor survival depending upon the environment. In the presence of interferons or generally a more TH-1 environment, it performs an antitumor activity and helps in immune clearance of the tumors, but in a more Th-2 cytokine-mediated environment, NF-κB plays the opposite role and helps in tumor survival and progression. The recent advances in immunotherapy and targeted therapy have offered a glimmer of hope in lung cancer treatment. It is also imperative to evaluate optimal combinatorial approaches, optimal drug sequencing, and redefining and streamlining clinical trials.
